# Exploring depressive symptom trajectories in COVID-19 patients with clinically mild condition in South Korea using remote patient monitoring: longitudinal data analysis

**DOI:** 10.3389/fpubh.2024.1265848

**Published:** 2024-04-10

**Authors:** Sumi Sung, Su Hwan Kim, Youlim Kim, Ye Seul Bae, Eui Kyu Chie

**Affiliations:** ^1^Department of Nursing Science, Research Institute of Nursing Science, Chungbuk National University, Cheongju, Chungcheongbuk-do, Republic of Korea; ^2^Department of Information Statistics, Gyeongsang National University, Jinju, Gyeongsangnam-do, Republic of Korea; ^3^Department of Radiation Oncology, Seoul National University College of Medicine, Seoul, Republic of Korea; ^4^Division of Healthcare Planning, Bigdata Research Institute, Kangbuk Samsung Hospital, Sungkyunkwan University School of Medicine, Seoul, Republic of Korea; ^5^Department of Family Medicine, Kangbuk Samsung Hospital, Sungkyunkwan University School of Medicine, Seoul, Republic of Korea; ^6^Institute of Radiation Medicine, Medical Research Center, Seoul National University, Seoul, Republic of Korea

**Keywords:** telehealth, telemedicine, depression, COVID-19, LCMM

## Abstract

**Background:**

During the height of the COVID-19 pandemic, the Korean government temporarily allowed full scale telehealth care for safety and usability. However, limited studies have evaluated the impact of telehealth by analyzing the physical and/or mental health data of patients with COVID-19 diagnosis collected through telehealth targeting Korean population.

**Objective:**

This study aimed to identify subgroup of depressive symptom trajectories in patients with clinically mild COVID-19 using collected longitudinal data from a telehealth-based contactless clinical trial.

**Methods:**

A total of 199 patients with COVID-19 were accrued for contactless clinical trial using telehealth from March 23 to July 20, 2022. Depressive symptoms were measured using the patient health questionnaire-9 on the start day of quarantine, on the final day of quarantine, and 1 month after release from quarantine. Additionally, acute COVID-19 symptoms were assessed every day during quarantine. This study used a latent class mixed model to differentiate subgroups of depressive symptom trajectories and a logistic regression model with Firth’s correction to identify associations between acute COVID-19 symptoms and the subgroups.

**Results:**

Two latent classes were identified: class 1 with declining linearity at a slow rate and class 2 with increasing linearity. Among COVID-19 symptoms, fever, chest pain, and brain fog 1 month after release from quarantine showed strong associations with class 2 (fever: OR, 19.43, 95% CI, 2.30–165.42; chest pain: OR, 6.55, 95% CI, 1.15–34.61; brain fog: OR, 7.03, 95% CI 2.57–20.95). Sleeping difficulty and gastrointestinal symptoms were also associated with class 2 (gastrointestinal symptoms: OR, 4.76, 95% CI, 1.71–14.21; sleeping difficulty: OR, 3.12, 95% CI, 1.71–14.21).

**Conclusion:**

These findings emphasize the need for the early detection of depressive symptoms in patients in the acute phase of COVID-19 using telemedicine. Active intervention, including digital therapeutics, may help patients with aggravated depressive symptoms.

## 1 Introduction

As the COVID-19 outbreak transition into a pandemic, the role and the use of telehealth is expanding and gaining significant background. Many countries have introduced remote patient monitoring systems that use telehealth to manage patients at remote locations (1–3). Telehealth has been shown to be an excellent method for delivering care as it allows not only patients but also health care providers to protect themselves from the risk of infection (1, 4). Since the outbreak of the COVID-19 pandemic, telemedicine, which had been under limited pilot phase in Korea, was temporarily allowed in full scale for patients diagnosed with COVID-19. Until May 2023, all patients with COVID-19 were obligated to quarantine at home for 5–7 days during the acute clinical phase. Patients with COVID-19 received prescriptions for related acute respiratory symptoms via contactless consultations during the quarantine period.

Meanwhile, the impact of COVID-19 on mental health has been continuously reported. The prevalence of depression symptoms in adults in the US during the COVID-19 pandemic was more than three times that of pre-pandemic era (5). In the UK, the prevalence of depression increased to 32% from 4.12% in the pre-pandemic period (6). In China, the prevalence of depression has been moderately high (7). South Korea likewise saw an increase in manifestation of depression. During the COVID-19 pandemic, 30.7% of 2,288 adult residents reported a Patient Health Questionnaire-2 score of 3, indicating a high prevalence of depression (8).

With the transition to the long-COVID era, many studies have explored mental health trajectories, including low, moderate, severe, and worsening mental health, using longitudinal data (9–11). Contributing social and psychological factors have also been identified. The unpredictable disease course of COVID-19 and COVID-19-related financial and social impairments have been reported to be related to the initial elevation in the level of depressive or anxiety symptoms (11–13). Moreover, the exacerbation of COVID-19 symptoms may also contribute to the development of mental health symptoms (14, 15).

Depression is strongly associated with somatic symptoms (16). Meanwhile, acute COVID-19-related somatic symptoms are associated with the exacerbation of depression and anxiety (15). In Korea, depression has been identified as the main symptom among COVID-19-related persistent symptoms (17). However, no study has explored the association between the trajectories of depression in the acute phase and acute COVID-19-related symptoms for these patients to provide a Korean perspective.

Against this background, the SMILE (Smart Monitoring solution for Infectious disease management through Lifestyle Evaluation) research team at Seoul National University Hospital established a remote patient monitoring system to effectively respond to the infectious disease. A contactless clinical trial protocol using telehealth was developed (18). Longitudinal data, including physical and mental health-related data, were prospectively collected from patients with COVID-19. Obvious next step was to demonstrate the impact of telehealth by analyzing the collected data. Thus, goal of current study was to identify subgroups of depressive symptom trajectories in patients with clinically mild COVID-19 in Korea and explore the contributing COVID-19-related symptoms to those groups using collected longitudinal data from a contactless clinical trial using telehealth.

## 2 Methods

### 2.1 Study design

This is a prospective observational study.

### 2.2 Participants and procedures

After institutional review board approval (IRB number: H-2107-049-1233), 199 adult patients with confirmed COVID-19 diagnosis quarantined at home were enrolled in this prospective trial from March 23 to July 20, 2022. The inclusion criteria were as follows: (1) 19 years or older; (2) confirmed COVID-19 diagnosis; (3) understand the study purpose, and (4) agree to participate in the trial.

This study was based on a published protocol developed for patients quarantined at residential treatment centers (18) with modification for home use. The study participants were recruited using convenience sampling. The research team displayed a poster that provided information on the research purpose and methods on the staff portal site and notice board at the study hospital. Patients who were hospital employees or acquaintances voluntarily contacted the research assistants and filled out an application form online through a URL or QR code in the poster.

Research assistants explained the purpose of the study to the prospective participants and oral informed consent was obtained over a phone call. Data were collected using Google Forms (Google, CA, USA). Through a mobile messenger, the study team sent the URL of the Google form containing an online questionnaire on mental health status. The participants completed online questionnaires on every quarantine day and 1 month after the release from quarantine.

### 2.3 Measures

Depression symptoms were measured using the patient health questionnaire-9 (PHQ-9) (19) at three time points: on the start day of quarantine (Time 1), on the final day of quarantine (Time 3), and 1 month after release from quarantine (Time 4). Participants were asked on the frequency of the nine potentially bothering symptoms. Each item was scored using the following four-point Likert scale: not at all = 0, several days a week = 1, more than half the week = 2, nearly every day = 3. Severity of depression was divided into five tiers, a score of 1–4 as none, 5–9 as mild, 10–14 as moderate, 15–19 as moderately severe, and 20–27 as severe.

General characteristics of the participants, including gender, age, past medical history, smoking status, and initial neuropsychiatric symptoms, were assessed at Time 1. Past medical history included history of diabetes mellitus, hypertension, cardiovascular disease, and respiratory disease. Initial neuropsychiatric symptoms included depression, lethargy, anxiety, sensitivity, insomnia, panic attack, and suicide attempts. Self-reported acute COVID-19 symptoms were assessed at every quarantine day (Time 2) and at Time 4. Self-reported acute COVID-19 symptoms at Time 2 included cough, sputum, fever, rhinorrhea, sore throat, dyspnea, chest pain, pain, sleeping difficulty, loss of smell, loss of taste, and various gastrointestinal symptoms, which includes nausea, vomiting, abdominal discomfort, constipation, and diarrhea.

### 2.4 Statistical analysis

All analyses were conducted using R version 4.2 (R Project for Statistical Computing). To identify the subgroups of depressive symptom trajectories over time, we used the latent class mixed model (LCMM), also called growth mixture modeling (20–23), subsequently selecting the models that provided the best fit for the data. To assess the clinical characteristics of the subgroups identified at Time 1, we conducted a logistic regression with subgroups identified as dependent variables and sociodemographic features, past medical history, initial psychological symptoms, and acute COVID-19 symptoms measured at Time 1 as independent variables. To support the sample size considerations for binary logistic regression analysis, we fitted the logistic regression model using a modified estimation procedure known as Firth’s correction (24). We then selected variables at Time 1 that were significantly associated with subgroup identification. Next, after selecting the clinical characteristics from Times 1, 2, and 4 associated with the subgroups of trajectories, we conducted additional logistic regression using a stepwise approach with subgroups identified as dependent variables and those variables measured at Times 2 and 4 as independent variables. In this analysis, we added previously selected significant variables at Time 1 to the logistic regression model as fixed variables. The model was also fitted using Firth’s correction (24), and statistical significance was tested at the *α* = 0.05 level.

## 3 Results

Table 1 presents the clinical characteristics of the 199 participants.

**Table 1:** Clinical characteristics of the participants.

Characteristics, *n* (%)		Time 1	Time 2	Time 4
**Gender**				
Male	63 (31.7)			
Female	136 (68.3)			
**Age (years), mean (SD)**	36.7 (9.4)			
**Age group (years)**				
Under 30	47 (23.6)			
30–39	89 (44.7)			
Over 40	63 (31.7)			
**Past medical history**				
Non-psychiatric	19 (9.5)			
Sleep disorder	20 (10.1)			
Neuropsychiatric treatment	22 (11.1)			
**Smoking status**				
Never-smoker	158 (79.4)			
Ex-smoker	25 (12.6)			
Smoker	16 (8.0)			
**Initial neuropsychiatric symptoms**				
Yes		135 (67.8)		
No		64 (32.2)		
**COVID-19 related symptoms**				
Cough		158 (79.4)	185 (93.0)	120 (60.3)
Sputum		148 (74.4)	189 (95.0)	96 (48.2)
Fever		80 (40.2)	87 (43.7)	9 (4.5)
Rhinorrhea		94 (47.2)	154 (77.4)	39 (19.6)
Sore throat		163 (81.9)	169 (84.9)	31 (15.6)
Dyspnea		5 (2.5)	14 (7.0)	20 (10.1)
Chest pain		16 (8.0)	26 (13.1)	9 (4.5)
Gastrointestinal symptoms		68 (34.2)	103 (51.8)	49 (24.6)
Pain		107 (53.8)	142 (71.4)	21 (10.6)
Sleeping difficulty		50 (25.1)	64 (32.2)	38 (19.1)
Loss of smell			46 (23.1)	39 (19.6)
Loss of taste			44 (22.1)	36 (18.1)
**Post-COVID-19-related symptoms**				
Headache				32 (16.1)
Brain fog				52 (26.1)
Fatigue				135 (67.8)

**Figure 1:**
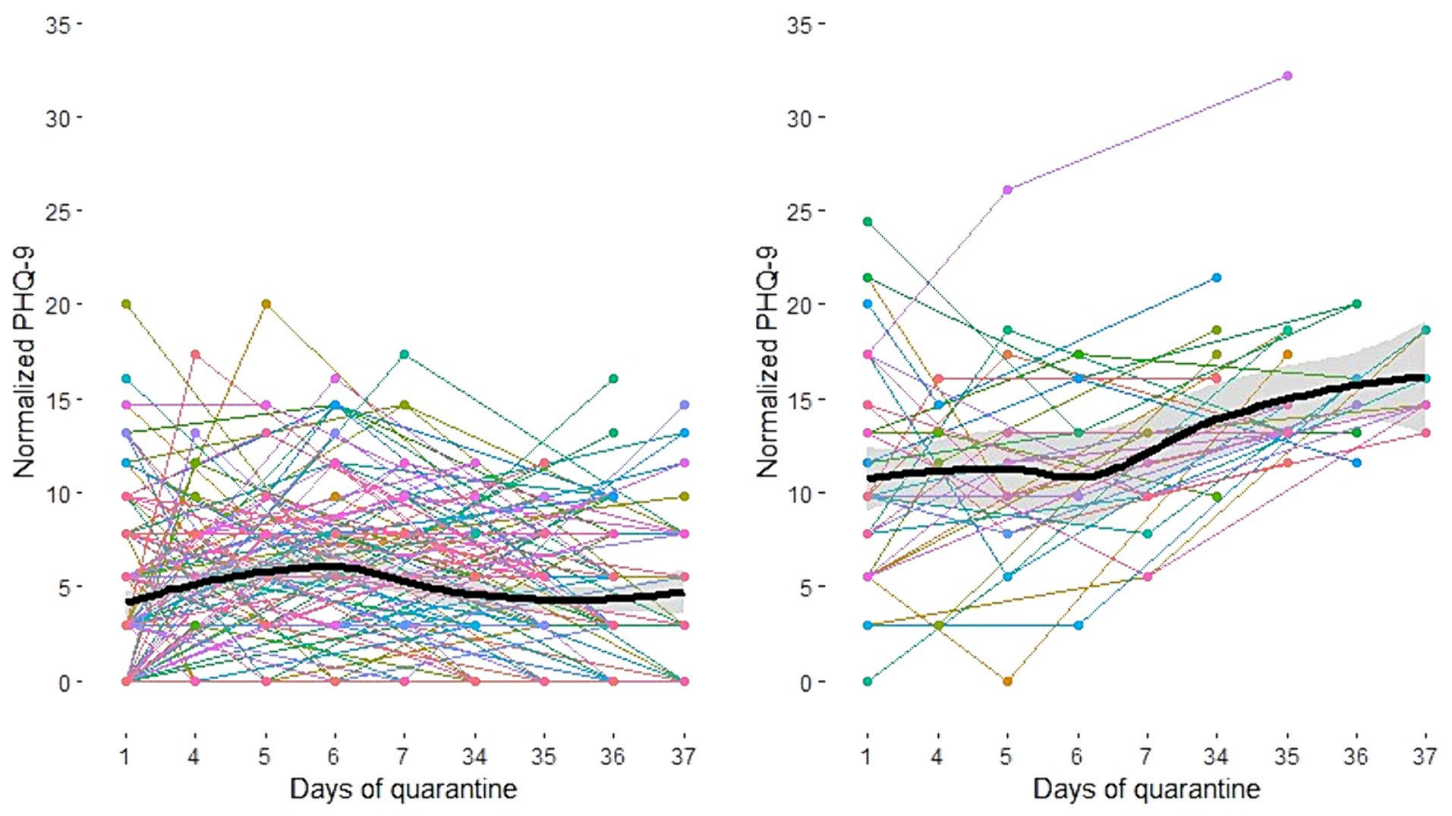
Estimated mean trajectories of depression symptoms in patients with COVID-19 (**Left**: Class 1, **Right**: Class 2).

The LCMM identified two latent classes as the best fit for the data. The estimated mean trajectories of the two classes are shown in Figure 1. In class 1 (*n* = 163; 81.9%), the mean trajectory declined linearly at a slow rate. The PHQ-9 mean scores of class 1 at each time point were 1.64 (SD 1.89) at time 1, 2.33 (SD 2.15) at time 3, and 1.62 (SD 1.68) at time 4. In class 2 (*n* = 36, 18.1%), patients had a higher level of depression symptoms than those in class 1 at time 1 and showed increased linearity with the progress of the quarantine period. The PHQ-9 mean scores of class 2 at each time point were 5.0 (SD 3.58) at time 1, 5.03 (SD 2.95) at time 3, and 7.89 (SD 2.57) at time 4.

Table 2 presents the clinical characteristics of the participants in the subgroups identified by the LCMM at time 1. Initial neuropsychiatric symptoms were observed in 80.6% of patients in class 2 and in 65% of patients in class 1, which showed statistically significant difference (*p* < 0.001). More than 70% of the patients in both classes experienced cough, sputum, and sore throat, among the COVID-19-related symptoms. Of note, there was statistically significant difference in incidence of chest pain (5.5 and 19.4% of patients in classes 1 and 2, respectively).

**Table 2:** Clinical characteristics of the participants at Time 1 in subgroups.

Characteristics	Class 1 (*n* = 163)	Class 2 (*n* = 36)	Total (*n* = 199)	$\chi^{2}$ /t	*p*
**Gender, *n* (%)**					
Male	53 (32.5)	10 (27.8)	63 (31.7)	0.13	0.72
Female	110 (67.5)	26 (72.2)	136 (68.3)		
**Age (years), mean (SD)**	37.2 (9.1)	34.4 (10.3)	36.7 (9.4)	−1.65	0.10
**Age group (years), *n* (%)**					
Under 30	33 (20.2)	14 (38.9)	47 (23.6)	5.68	0.06
30–39	76 (46.6)	13 (36.1)	89 (44.7)		
Over 40	54 (33.1)	9 (25.0)	63 (31.7)		
**Past medical history, *n* (%)**					
Non-psychiatric	17 (10.4)	2 (5.6)	19 (9.5)	0.34	0.56
Sleep disorder	13 (8.0)	7 (19.4)	20 (10.1)	3.12	0.08
Neuropsychiatric treatment	15 (9.2)	7 (19.4)	22 (11.1)	2.19	0.14
**Smoking status, *n* (%)**					
Never-smoker	131 (80.4)	27 (75.0)	158 (79.4)	0.68	0.71
Ex-smoker	20 (12.3)	5 (13.9)	25 (12.6)		
Smoker	12 (7.4)	4 (11.1)	16 (8.0)		
**Initial neuropsychiatric symptoms**					
Yes	106 (65.0)	29 (80.6)	135 (67.8)	20.41	<0.001
No	57 (35.0)	7 (19.4)	64 (32.2)		
**COVID-19-related symptoms**					
Cough	130 (79.8)	28 (77.8)	158 (79.4)	<0.001	0.97
Sputum	119 (73.0)	29 (80.6)	148 (74.4)	0.53	0.47
Fever	63 (38.7)	17 (47.2)	80 (40.2)	0.58	0.45
Rhinorrhea	72 (44.2)	22 (61.1)	94 (47.2)	2.75	0.10
Sore throat	135 (82.8)	28 (77.8)	163 (81.9)	0.22	0.64
Dyspnea	4 (2.5)	1 (2.8)	5 (2.5)	<0.001	1.0
Chest pain	9 (5.5)	7 (19.4)	16 (8.0)	5.96	0.02
Gastrointestinal symptoms	52 (31.9)	16 (44.4)	68 (34.2)	1.54	0.21
Pain	89 (54.6)	18 (50.0)	107 (53.8)	0.10	0.75
Sleeping difficulty	37 (22.7)	13 (36.1)	50 (25.1)	2.15	0.14

Table 3 presents the results of the logistic regression model for COVID-19-related symptoms at time 1. Among COVID-19 symptoms, rhinorrhea and chest pain showed positive associations with increased odds of exacerbation of depression. Although extent was not statistically significant (rhinorrhea: odds ratio [OR], 2.06, 95% CI, 0.96–4.57; chest pain: OR, 3.07, 95% CI, 0.92–10.31), these two variables were chosen as fixed variables for the final model.

**Table 3:** Association between COVID-19 symptoms at Time 1 and depression Class 2.

Characteristics	Odds ratio	95% Confidence interval	*p*
**Gender**			
Male			
Female	1.46	0.59–3.91	0.42
**Age group (years)**			
Under 30			
30–39	0.53	0.22–1.30	0.16
Over 40	0.43	0.15–1.16	0.097
**Past medical history**			
Non-psychiatric	0.70	0.11–3.22	0.66
Sleep disorder	1.85	0.59–5.45	0.28
Neuropsychiatric treatment	1.54	0.51–4.30	0.43
**Initial neuropsychiatric symptoms**			
Yes			
No	1.37	0.56–3.63	0.50
**Smoking status**			
Never-smoker			
Ex-smoker	1.26	0.37–3.83	0.70
Smoker	2.24	0.50–8.95	0.28
**COVID-19-related symptoms at Time 1**			
Cough	0.67	0.27–1.77	0.40
Sputum	1.22	0.51–3.20	0.67
Fever	1.12	0.50–2.47	0.79
Rhinorrhea	2.06	0.96–4.57	0.064
Sore throat	0.67	0.24–2.06	0.47
Dyspnea	0.55	0.04–4.42	0.59
Chest pain	3.07	0.92–10.31	0.068
Gastrointestinal symptoms	1.45	0.66–3.16	0.65
Pain	1.14	0.49–2.68	0.76
Sleeping difficulty	1.19	0.48–2.77	0.70

Table 4 presents the association between COVID-19 symptoms and the class 2. The final model included rhinorrhea and chest pain at time 1; cough at time 2; and fever, rhinorrhea, chest pain, gastrointestinal symptoms, sleeping difficulty, headache, and brain fog at time 4. Among these symptoms, fever at time 4 showed a strong association with class 2, indicating the exacerbation of depression during quarantine (OR, 19.43; 95% CI, 2.30–165.42). Patients with chest pain or brain fog at time 4 were more likely to have exacerbated depression during quarantine (chest pain: OR, 6.55, 95% CI, 1.15–34.61; brain fog: OR, 7.03, 95% CI, 2.57–20.95). In addition, patients who experienced gastrointestinal symptoms or sleeping difficulty at time 4 were more likely to have exacerbated depression during quarantine (gastrointestinal symptoms: OR, 4.76, 95% CI, 1.71–14.21; sleeping difficulty: OR, 3.12, 95% CI, 1.71–14.21).

**Table 4:** Association between COVID-19 symptoms and depression Class 2.

Characteristics	Odds ratio	95% Confidence interval	*p*
**Gender**			
Male			
Female	0.67	0.21–2.13	0.49
**Age group (years)**			
Under 30			
30 to 39	0.64	0.18–2.28	0.48
Over 40	0.56	0.15–2.10	0.39
**COVID-19-related symptoms at Time 1**			
Rhinorrhea	1.30	0.48–3.51	0.61
Chest pain	3.81	0.81–17.36	0.09
**COVID-19-related symptoms at Time 2**			
Cough	0.29	0.07–1.24	0.09
**COVID-19-related symptoms at Time 4**			
Fever	19.43	2.30–165.42	0.007
Rhinorrhea	2.97	1.05–8.46	0.039
Chest pain	6.55	1.15–34.61	0.035
Gastrointestinal symptoms	4.76	1.71–14.21	0.002
Sleeping difficulty	3.12	1.05–9.69	0.041
Headache	2.39	0.74–7.75	0.14
Brain fog	7.03	2.57–20.95	<0.001

## 4 Discussion

Longitudinal data related to depressive symptoms and COVID-19-related symptoms was collected through a contactless clinical trial using telehealth targeting patients with clinically mild symptoms at the acute phase of COVID-19 in South Korea. The results showed two subgroups of depressive symptom trajectories from COVID-19 infection to 1 month after quarantine: the stable group (class 1) and the exacerbated group (class 2).

LCMM was used to identify the two subgroups of patients with COVID-19 based on the trajectory of their PHQ-9 scores across multiple time points. Studies have used LCMM to successfully identify meaningful latent subgroups using longitudinal data (25). For example, it has been used to categorize trajectories of depression and anxiety symptom changes (26) and shown to perform as accurately as the traditional cutoff score approach in identifying heterogeneous subgroups in a longitudinal study on perinatal depression (27). The LCMM provided strong robustness in current analysis given the lack of a traditional or standard method for categorizing longitudinal post-COVID-19 depression changes.

Among the acute COVID-19-related symptoms at time 4, chest pain, gastrointestinal symptoms, sleeping difficulty, and brain fog, which are commonly observed in patients with depression (16), were significantly associated with class 2. The Patient Health Questionnaire 15, which is one of the most useful tools for measuring somatization in psychiatric patients, also includes the following symptoms: trouble sleeping, chest pain, and gastrointestinal symptoms, such as constipation, loose bowels, diarrhea, nausea, and indigestion (28). Indeed, 69% of patients with major depressive disorder who visited primary care facilities reported that physical symptoms were the main reason for their hospital visit (16). Ran et al. (29) found a significant correlation between depression and somatization in patients with COVID-19, which is consistent with the findings of the current study.

In addition, patients from Eastern cultures, compared to Western counterparts, tend to deny psychological symptoms and complain more about physical symptoms (16, 30–32). Therefore, early detection of depression is important for patients with persistent non-specific somatic symptoms, even after recovery from COVID-19. In Eastern cultures such as Korea, health authorities should prepare public health measures to monitor not only the progress of infectious diseases, but also the mental health.

The current study attempted to monitor and analyze acute COVID-19 somatic and depressive symptoms simultaneously using telehealth services for patients in remote locations. Results suggest that early detection of patients with depressive symptoms in the acute phase of COVID-19 using telemedicine is feasible. Digital therapeutics have been reported to be effective for patients with mental illnesses (33, 34). During the COVID-19 pandemic, even the general population without a history of mental illness preferred digital therapeutics to visiting psychiatric clinics (35). Thus, worsening of depressive symptoms may be prevented by offering digital therapeutics intervention to those with early detection during quarantine, when visit to the clinic in person can be limited (36).

### 4.1 Limitations

First, patient accrual done using convenience sampling may have led to the snowball sampling. Thus, the generalizability of the study results cannot be ensured. Second, the data were assessed using patient self-reports, which may be led to inaccurate estimates of symptom changes. Third, the patients could have undergone interventions, such as drug therapy for COVID-19 symptoms, that were not considered in the statistical analyses. Fourth, the PHQ-9 measures depressive symptoms over the past 2 weeks, resulting in timeline issues for Times 1 and 3 that must be considered with respect to the quarantine duration of approximately 7 days. Further study may be conducted using more specific tools such as the Hamilton depression rating scale (37) rather than PHQ-9. Fifth, current study did not collect social determinants and socio-economic status of the participants, which play an important role in mental health, but was not considered in the current analysis.

## 5 Conclusion

Results from current study demonstrated the potential impact of telehealth through the use of longitudinal data collected from a contactless clinical trial. We identified two subgroups of depressive symptom trajectories in Korean patients with clinically mild COVID-19: the stable group (class 1) and the worsening group (class 2). The COVID-19-related symptoms associated with these groups were fever, chest pain, brain fog, sleeping difficulty, and gastrointestinal symptoms 1 month after release from quarantine. Findings from current analysis suggested that early detection of patients with high risk may provide a chance for more effective intervention, such as digital therapeutics, prior to deterioration of mental health. In addition, further study may help to elucidate how post-COVID-19 syndrome impacts mental health of patients with COVID-19, and which of social determinants of health, such as socioeconomic status, education level, and ethnicity of these patients impact mental health to be nominated as potential risk factor.

## Data availability statement

The datasets presented in this article are not readily available because hospital regulation restrictions and patient privacy concerns. Requests to access the datasets should be directed to EC, ekchie93@snu.ac.kr.

## Ethics statement

The studies involving humans were approved by Institutional Review Board at Seoul National University Hospital (IRB number: H-2107-049-1233). The studies were conducted in accordance with the local legislation and institutional requirements. Written informed consent from the patients/participants was not required to participate in this study in accordance with the national legislation and the institutional requirements.

## Author contributions

SS: Conceptualization, Writing – review & editing, Formal analysis, Methodology, Writing – original draft. SK: Formal analysis, Methodology, Writing – original draft, Writing – review & editing. YK: Formal analysis, Writing – review & editing. YB: Formal analysis, Writing – original draft, Writing – review & editing, Conceptualization, Methodology. EC: Conceptualization, Writing – review & editing, Funding acquisition, Supervision.
